# SufA – a bacterial enzyme that cleaves fibrinogen and blocks fibrin network formation

**DOI:** 10.1099/mic.0.021311-0

**Published:** 2009-01

**Authors:** Christofer Karlsson, Matthias Mörgelin, Mattias Collin, Rolf Lood, Marie-Louise Andersson, Artur Schmidtchen, Lars Björck, Inga-Maria Frick

**Affiliations:** 1 Division of Infection Medicine, Department of Clinical Sciences, Lund University, BMC B14, 221 84 Lund, Sweden; 2 Division of Dermatology and Venereology, Department of Clinical Sciences, Lund University, BMC B14, 221 84 Lund, Sweden

## Abstract

*Finegoldia magna* is a member of the normal human bacterial flora on the skin and other non-sterile body surfaces, but this anaerobic coccus is also an important opportunistic pathogen. SufA was the first *F. magna* proteinase to be isolated and characterized. Many bacterial pathogens interfere with different steps of blood coagulation, and here we describe how purified SufA efficiently and specifically cleaves fibrinogen in human plasma. SufA is both secreted by *F. magna* and associated with the bacterial surface. Successful gene targeting has previously not been performed in anaerobic cocci, but in order to study the role of the SufA that is present at the bacterial surface, we constructed an *F. magna* mutant that expresses a truncated SufA lacking proteolytic activity. In contrast to wild-type bacteria that delayed the coagulation of human plasma, mutant bacteria had no such effect. Wild-type and mutant bacteria adhered to keratinocytes equally well, but in a plasma environment only wild-type bacteria blocked the formation of fibrin networks surrounding adherent bacteria. The effective cleavage of fibrinogen by SufA suggests that the interference with fibrin network formation represents an adaptive mechanism of *F. magna* with potential implications also for pathogenicity.

## INTRODUCTION

The Gram-positive anaerobic coccus *Finegoldia magna* is part of the human commensal flora colonizing the human skin, oropharynx, and gastrointestinal and urogenital tracts (Murdoch, 1998). It is also an opportunistic pathogen and the most commonly isolated species among Gram-positive anaerobic cocci in clinical specimens (Murdoch, 1998). Typical infections caused by *F. magna* are soft tissue infections, wound infections, bone and joint infections and vaginosis (Bowler & Davies, 1999; Hansson *et al.*, 1995; Murdoch & Shah, 1999; Stephens *et al.*, 2003). SufA (Subtilase of *Finegoldia magna*) is the first described proteinase of *F. magna*. This serine proteinase is associated with the bacterial cell surface, and is also found in substantial amounts in the bacterial growth medium. SufA cleaves and inactivates the antibacterial peptide LL-37 and the chemokine MIG/CXCL9, which also has antibacterial activity (Egesten *et al.*, 2007; Karlsson *et al.*, 2007), and homologues of SufA are present in the majority of *F. magna* strains (Karlsson *et al.*, 2007), including the recently published genome of *F. magna* ATCC 29238 (Goto *et al.*, 2008).

Many pathogenic bacteria interact with and manipulate the host coagulation system by activating or repressing homeostatic factors through proteolytic degradation or protein binding (for a review see Sun, 2006). Fibrinogen is the major clotting protein of human plasma, playing key roles in coagulation and homeostasis. Upon vascular injury, fibrin polymerization and clotting are initiated by thrombin cleavage of fibrinogen into fibrin. Fibrinogen consists of three pairs of non-identical chains, A*α*, B*β* and *γ*, linked together with 29 disulfide bonds in a complex of 340 kDa (Mosesson, 2005). Several bacterial pathogens, *Streptococcus pyogenes*, *Pseudomonas aeruginosa*, *Porphyromonas gingivalis* and *Treponema denticola*, produce fibrinogen-degrading proteinases (for references see Travis & Potempa, 2000). In addition, a cysteine proteinase of *Staphylococcus epidermidis* (Oleksy *et al.*, 2004) and subtilases of group B streptococci (Harris *et al.*, 2003) and the fungus *Acremonium* (Liu *et al.*, 2007) have been reported to cleave fibrinogen, suggesting that this mechanism plays an important role in many host–microbe relationships.

In the present study we demonstrate that purified and soluble SufA efficiently cleaves fibrinogen in plasma, and that SufA associated with the surface of *F. magna* prevents the formation of fibrin networks. We also describe the first example of gene targeting in a Gram-positive anaerobic coccus.

## METHODS

### Proteins, bacterial strains and growth conditions.

Human serum albumin and fibrinogen were purchased from Sigma. *Finegoldia magna* strain ALB8 isolate was from Lund University Hospital, Lund, Sweden and has been described earlier (de Château & Björck, 1994). Bacteria were grown under strict anaerobic conditions (Anaerobic Workstation, Elektrotek) in Todd–Hewitt broth (TH, Difco) supplemented with 0.5 % (v/v) Tween-80. For cultivation of *F. magna* mutant CK05, 200 μg kanamycin ml^−1^ was added. *Escherichia coli* TOP10 was purchased from Invitrogen and cultured in Luria broth (LB, Difco). Recombinantly expressed SufA is described elsewhere (Karlsson *et al.*, 2007). For native SufA purification, *F. magna* bacteria were treated with papain, and SufA was purified as described earlier (Karlsson *et al.*, 2007). Briefly, following the digestion, papain was inactivated by adding E64, the supernatant was collected by centrifugation and papain was removed by dialysis. The proteins were then separated by ion-exchange chromatography and fractions containing SufA were further purified by gel filtration. Fractions were analysed by SDS-PAGE, Western blotting using antibodies against recombinant SufA as probe, and gelatin zymography. Also, crude papain digests and concentrated growth medium from ALB8 and the mutant CK05 were analysed by Western blotting and gelatin zymography.

### SDS-PAGE, mass spectrometry, immunoblotting and zymography.

SDS-PAGE was performed as described by Laemmli (1970) using a total polyacrylamide concentration of 8 % or 10 %, and 3.3 % cross-linking. Gels were stained with Coomassie R-250 or separated proteins were transferred onto an Immobilon-P membrane (Millipore) by using the Mini Trans-Blot system (Bio-Rad). SufA was detected with antibodies raised against recombinant SufA (1 : 1000) (Karlsson *et al.*, 2007). Human tissue factor monoclonal antibodies were purchased from American Diagnostica. The monoclonal antibody against bovine osteonectin/BM-40, developed by Dr John D. Termine, was obtained from the Developmental Studies Hybridoma Bank developed under the auspices of the NICHD and maintained by The University of Iowa, Department of Biological Sciences, Iowa City, IA 52242, USA. Bound antibodies were detected by using horseradish peroxidase-conjugated goat anti-rabbit IgG or goat anti-mouse IgG (Bio-Rad) and Supersignal West Pico chemiluminescent substrate (Pierce).

Coomassie R-250-stained bands of interest were excised from SDS-PAGE gels and analysed by matrix-assisted laser desorption/ionization-time of flight mass spectrometry (MALDI-TOF MS). Briefly, the excised protein bands were destained and enzymically digested with trypsin (Promega). The tryptic peptides were then spotted onto a MALDI target plate. MALDI-MS mass spectra were acquired on a MALDI micro MX mass spectrometer (Waters) followed by automated protein identification by protein mass fingerprinting searching the Swiss-Prot database, limited to *Homo sapiens*, with the search engine MASCOT (http://www.matrixscience.com). The following matcher parameters were used: constant modification of cysteine by carbamidomethylation, variable modification of methionine by oxidation. MALDI-TOF was carried out at the SCIBLU proteomics resource centre at Lund University (http://www.lth.se/sciblu/).

For zymography, protein samples were dissolved in non-reducing sample buffer and incubated for 5 min at room temperature. Proteins were separated by SDS-PAGE using 8 % polyacrylamide gels containing 0.1 % porcine gelatin (Bio-Rad). After electrophoresis, gels were incubated in 2.5 % Triton X-100 for 30 min at 37 °C, washed in 50 mM Tris/HCl pH 7.5, 0.2 M NaCl and 5 mM CaCl_2_ and then incubated for 18 h in the same buffer. Areas of proteolytic activity were detected by Coomassie R-250 staining of the gel (Liotta & Stetler-Stevenson, 1990).

### *F. magna* transformation.

The transformation protocol used for *Streptococcus pyogenes* (Simon & Ferretti, 1991) was modified for *F. magna*. A 10 ml solution of stationary-phase bacteria was centrifuged and washed with water, followed by 0.5 M sucrose, and then resuspended in 0.1 ml 0.5 M sucrose. To 100 μl of the bacterial cell solution 5 μg DNA was added and the bacteria were electroporated using the Gene Pulser II system (Bio-Rad) with settings 2.3 kV, 3 μF, 800 Ω in 2 mm cuvettes. Electroporated bacteria were incubated in 10 ml TH medium, supplemented with 0.5 % Tween-80 and 10 % fetal calf serum, for 18 h in an anaerobic environment for phenotypic expression. Appropriate antibiotics were added to the bacterial culture for selection (100 μg kanamycin ml^−1^) and incubation was continued for 3 days. The culture was then plated on TH-agar plates containing the same antibiotic as above.

### Mutagenesis of *sufA* in *F. magna* ALB8.

For insertional gene disruption of *sufA*, plasmid pFW13 (GenBank accession number U50978) was used for genomic integration of a truncated *sufA* gene. A PCR product covering bp 90–1100 of the *sufA* coding sequence (CDS) (GenBank accession number DQ679960) was amplified from *F. magna* ALB8 genomic DNA using primers forward 5′-CTCGAGGATAGCACTACATATGCCAAACTTCA-3′ and reverse 5′-AAGCTTGTCAGGGTTGATAGCTAAGTTAGTCTT-3′. The forward primer incorporated a *Xho*I restriction site and the reverse included a *Hin*dIII site (underlined). The product was cloned into pCR4-Topo vector (Invitrogen) according to the manufacturer's protocol. The pCR4-Topo : sufA vector was digested with *Xho*I and *Hin*dIII. The 1010 bp *sufA* fragment was ligated into *Xho*I- and *Hin*dIII-digested pFW13. The ligation was transformed into *E. coli* TOP10 cells (Invitrogen) and transformants were selected for kanamycin resistance. Purified pFW13-sufA plasmid was then used for electroporation of *F. magna* ALB8 bacteria (see above). Mutants were analysed using PCR with the following primer combinations: PCR1 forward 5′-CAAGGCGTAAAGGCAGACCAAC-3′ and reverse 5′-CCTCCTTTTGGTTACCTCAC-3′; PCR2 forward 5′-TCTTTTCTACGGGGTCTGAC-3 and reverse 5′-CAGTAGCATCAATGGAAAATACAA-3′; PCR3 forward 5′-CAAGGCGTAAAGGCAGACCAAC-3′ and reverse 5′-TCTACTGTAACCTGAACCCATTCCC-3′. Crude protein extracts and concentrated growth media of the mutants were analysed using antibodies against recombinant SufA and with gelatin zymography. One clone, designated CK05, was chosen for further analysis with DNA sequencing and Southern blotting.

### Southern blots.

*F. magna* chromosomal DNA was isolated by using the Gentra Puregene kit with the following modification: the bacteria were incubated with 1 U mutanolysin (Sigma) per mg cells in TE-buffer (10 mM Tris/HCl pH 7.5 and 1 mM EDTA) at 37 °C for 18 h before cell lysis.

Internal fragments of the *sufA* (bps 39–495 of the CDS) gene and the pFW13 kanamycin-resistance gene *aacA/aphD* (bps 346–822 of the CDS) to be used as Southern blot probes were cloned as follows: ALB8 chromosomal DNA was amplified with primers forward 5′-TTGTTTTCATTGGCATTACC-3′ and reverse 5′-CAAGGCTGATACTTTGTGGG-3′, and plasmid pFW13 was amplified with primers forward 5′-ATACAGAGCCTTGGGAAGAT-3′ and reverse 5′-GGTAGTGGTTATGATAGTGTGGCA-3′. The generated PCR products were purified and biotin-labelled using the Biotin DecaLabel kit (Fermentas). Three micrograms of chromosomal DNA from ALB8 and CK05 was digested with *Eco*RI and separated on agarose gel. The hybridization signals were detected with the Biotin Chromogenic Detection kit (Fermentas) according to the standard protocol recommended by the manufacturer.

### Clotting assays.

Fibrinogen polymerization was measured in a coagulometer (Amelung). SufA purified from the cell surface of the ALB8 strain (Karlsson *et al.*, 2007) or bacterial suspensions (30 μl of 10^8^, 5×10^8^ or 10^9^ c.f.u. in 13 mM sodium citrate buffer) was incubated with 100 μl citrate-treated plasma (Lund University Hospital) or fibrinogen (Sigma) solution (3 mg ml^−1^ in 13 mM sodium citrate buffer) for 30 min at 37 °C. Samples incubated with bacteria were pelleted and 100 μl of the resulting supernatants was clotted by the addition of 100 μl thrombin clotting time (TCT) reagent (Haemoclot Thrombin Time, Hyphen Biomed). Clotting of supernatants from SufA-treated plasma was also initiated by the addition of 100 μl DAPTTIN (Technoclone) for 200 s at 37 °C followed by addition of 100 μl 25 mM CaCl_2_ (for activated partial thromboplastin time, aPTT) or 200 μl ThromboMAX with calcium (for prothrombin time, PT; Trinity Biotech). The concentration of fibrinopeptide A (FPA) following SufA cleavage of fibrinogen was measured by using a FPA ELISA kit from Haemochrom Diagnostica.

### Cell culture and adherence assay.

A human keratinocyte cell line (HaCaT) was used to test adherence of *F. magna*. Cells were cultured in serum-free medium (K-SFM) supplemented with 5 ng recombinant human epidermal growth factor (EGF) μl^−1^, 50 μg bovine pituitary extract ml^−1^ and 650 ng gentamicin ml^−1^ (all from Gibco-BRL, Invitrogen), at 37 °C in an atmosphere containing 5 % CO_2_ with 100 % relative humidity. Cells were grown to monolayer and confluence on Thermanox coverslips (Nunc) placed in a 24-well culture plate or directly in 24-well culture plates. Monolayers were washed three times with PBS and infected with 10^8^ c.f.u. bacteria for 2 h at 37 °C. Infected monolayers were washed three times with PBS to remove non-adherent bacteria and then incubated with citrate-treated plasma or PBS for 15 min at 37 °C. After washing as above, the cells were prepared for analysis by electron microscopy.

Keratinocyte proteins for immunoblotting were extracted from confluent monolayers with 20 % SDS buffer and boiling. Proteins in keratinocyte conditioned media were precipitated with acetone.

### Electron microscopy.

For scanning electron microscopy, cells and bacteria on coverslips were fixed in 2 % (v/v) glutaraldehyde, 0.1 M sodium cacodylate, 0.1 M sucrose, pH 7.2, for 1 h at 4 °C, and washed with 0.15 M cacodylate, pH 7.2. The samples were post-fixed with 1 % (w/v) osmium tetroxide, 0.15 M sodium cacodylate, pH 7.2, for 1 h at 4 °C, washed, and stored in cacodylate buffer. The samples were dehydrated with an ascending ethanol series (10 min per step), dried, mounted on aluminium holders, sputtered with palladium/gold, and examined in a JEOL JSM-350 scanning electron microscope.

For transmission electron microscopy, cells and bacteria in 24-well culture plates were harvested with a scraper and fixed for 1 h at room temperature and then overnight at 4 °C in 2.5 % glutaraldehyde in 0.15 M sodium cacodylate, pH 7.4 (cacodylate buffer). Samples were washed with cacodylate buffer and post-fixed for 1 h at room temperature in 1 % osmium tetroxide in cacodylate buffer, dehydrated in a graded series of ethanol, and then embedded in Epon 812 (SPI Supplies) using acetone as an intermediate solvent. Specimens were sectioned with a diamond knife into 50–70 nm thick ultrathin sections on an LKB ultramicrotome. The ultrathin sections were stained with uranyl acetate and lead citrate. Specimens were observed in a JEOL JEM 1230 electron microscope operated at 80 kV accelerating voltage. Images were recorded with a Gatan Multiscan 791 CCD camera.

For negative staining, 3 mg fibrinogen ml^−1^ was incubated with 30 nM SufA or PBS at 37 °C for 1 h. Samples were adsorbed onto a 400-mesh carbon-coated copper grid, which was rendered hydrophobic by glow discharge at low pressure in air. The grid was immediately blotted, briefly washed with two drops of water, and stained with 0.75 % uranyl formate for 15 s. Specimens were studied in a JEOL 1200 Ex transmission electron microscope operated at 60 kV accelerating voltage and ×75 000 magnification.

## RESULTS

### SufA cleaves fibrinogen in human plasma

**Fig. 1. f1:**
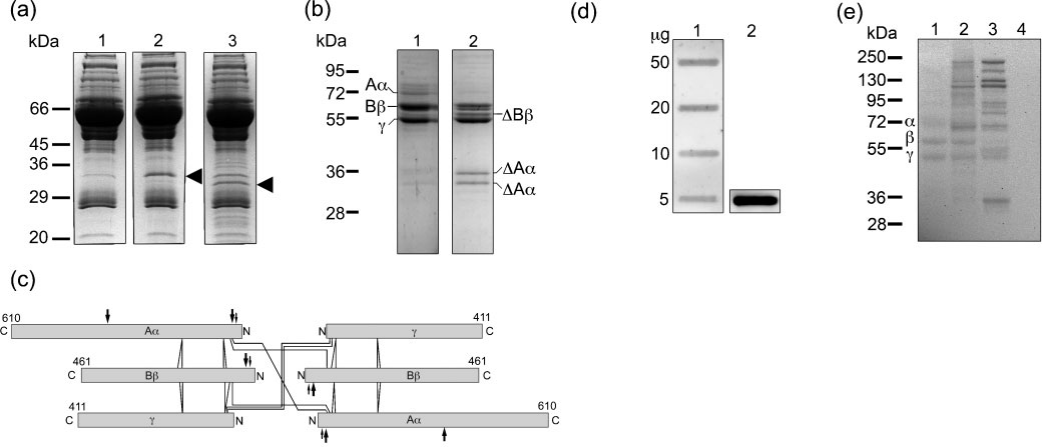
SufA degrades and binds fibrinogen. Human citrate-treated plasma or fibrinogen solution (3 mg ml^−1^) was incubated with SufA purified from the cell surface of *F. magna* and analysed by SDS-PAGE. Degradation products were identified by mass spectrometry. (a) A 10 μl aliquot of 5 % human plasma was incubated for 60 min at 37 °C with: lane 1, PBS; lane 2, 3 nM SufA; lane 3, 30 nM SufA. Degradation products in lanes 2 and 3, marked with an arrowhead, were identified by MALDI-TOF. (b) A 10 μg aliquot of human fibrinogen was incubated for 60 min at 37 °C with: lane 1, PBS; lane 2, 30 nM SufA. The positions of fibrinogen A*α*, B*β* and *γ* chains are shown in lane 1. Degradation products in lane 2 were identified by MALDI-TOF as indicated (Δ). (c) Schematic representation of fibrinogen with predicted SufA cleavage sites. The A*α*, B*β* and *γ* chains of fibrinogen are represented by bars proportional to the numbers of amino acid residues in each chain without the signal peptide. The NH_2_ and C termini are labelled N and C. Intrachain disulfide bonds are indicated with solid lines. Interchain disulfide bonds are not shown. Thrombin cleavage sites are indicated with small arrows and SufA cleavage sites are indicated with large arrows. (Adapted from Weisel, 2005.) (d) Various amounts of fibrinogen (lane 1) and 5 μg recombinant inactive SufA (lane 2) were applied in slots to a PVDF membrane. The membrane was incubated with recombinant inactive SufA (10 μg ml^−1^) for 18 h at 4 °C. Bound protein was detected with polyclonal rabbit antibodies raised against recombinant SufA. (e) A 10 μg aliquot of fibrinogen was incubated for 60 min at 37 °C with: lane 1, PBS; lane 2, 30 nM heat-inactivated SufA; lane 3, 30 nM active SufA. Following incubation, samples were separated by SDS-PAGE under reducing conditions and blotted to a PVDF membrane. Human serum albumin (10 μg) was used as a negative control (lane 4). The membrane was incubated with recombinant inactive SufA as in (d) and bound protein was detected with SufA antibodies.

In order to investigate whether SufA is proteolytically active on plasma proteins, the enzyme was purified from the cell surface of *F. magna* ALB8 as previously described (Karlsson *et al.*, 2007). SufA, at concentrations of 3 nM or 30 nM, was incubated for 1 h with human plasma, and two prominent degradation products with apparent molecular masses of 32 and 35 kDa were detected by SDS-PAGE (Fig. 1a[Fig f1], lanes 2 and 3). MS analysis of these protein bands identified both cleavage products as fibrinogen A*α*-chain fragments. All matched peptides were identified in the NH_2_-terminal region, suggesting that SufA may release a C-terminal fragment of the A*α* chain from the fibrinogen molecule (Table 1, samples 1 and 2). The cleavage of fibrinogen is rapid as judged from incubations of plasma and SufA for various time points, and after 5 min of incubation, degradation products could already be seen (data not shown). No other proteins could be identified in the protein bands using the MASCOT search engine and based on the probability-based Mowse scoring. The C-terminal A*α* chain is known to be particularly susceptible to proteolysis and lower forms are generally present in plasma fibrinogen, explaining the weak band of 35 kDa present in the PBS control (Fig. 1a[Fig f1], lane 1) (Weisel, 2005). To further confirm the fibrinogen-cleaving activity, purified fibrinogen was incubated with SufA and again the 32 and 35 kDa degradation products were generated (Fig. 1b[Fig f1]). In addition, a fragment of approximately 58 kDa appeared. Mass spectrometry identified the 58 kDa fragment as the B*β* chain of fibrinogen, with peptide masses matching sequences between residues 164 and 491 (including the absolute C terminus) (Table 1, sample 5). The two smaller fragments were identified as the A*α* chains, with matching peptides between residues 49 and 287 (Fig. 1b[Fig f1], Table 1, samples 3 and 4). Electron microscopy analysis following negative staining of fibrinogen cleaved by SufA revealed no visible differences as compared to untreated fibrinogen (data not shown), indicating that the overall structure of the molecule remains intact although portions of extending polypeptide chains are removed. A schematic depiction of the fibrinogen molecule with the predicted SufA cleavage sites is shown in Fig. 1(c). Other major human plasma proteins (albumin and IgG) were not degraded by SufA (data not shown). However, it cannot be excluded that less abundant plasma proteins are cleaved by the enzyme.

To investigate the interactions between fibrinogen and SufA, fibrinogen was directly applied to a PVDF membrane. The membrane was then incubated with a non-proteolytically active recombinantly expressed SufA (Karlsson *et al.*, 2007), followed by antibodies against SufA to detect bound protein. Fig. 1(d) shows that recombinant SufA interacts with native fibrinogen. Also the individual A*α*, B*β* and *γ* chains and their 58 kDa and 35 kDa degradation products, generated by SufA treatment, interact with the proteinase as demonstrated by Western blot analysis (Fig. 1e[Fig f1], lanes 1 and 3). The protein bands above 95 kDa in Fig. 1(e), lanes 2 and 3, represent various multimeric forms of SufA present in the SufA preparation (Karlsson *et al.*, 2007). Such size heterogeneity is a common property among surface proteins of Gram-positive bacteria, also following separation by SDS-PAGE (de Château *et al.*, 1996; Fischetti, 1989; Flores & Ferrieri, 1996). No binding was observed to human serum albumin (Fig. 1e[Fig f1], lane 4).

**Fig. 2. f2:**
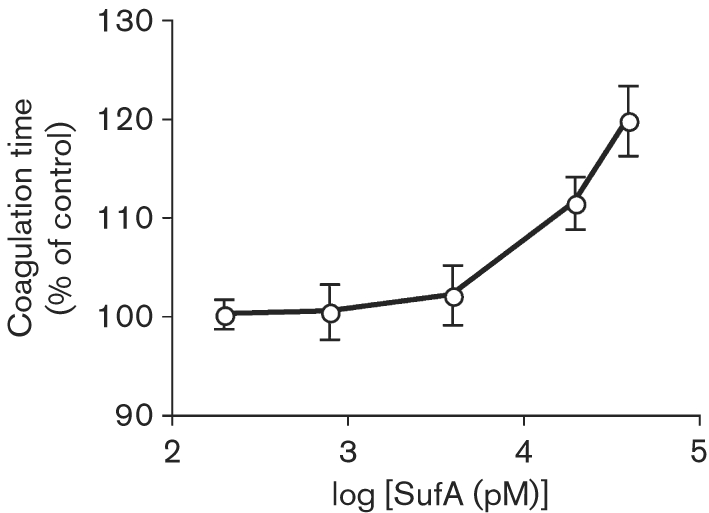
Effect of SufA on the clotting of plasma. Human citrate-treated plasma was incubated with purified SufA at the indicated concentrations or, as a control, with 13 mM sodium citrate buffer for 30 min at 37 °C. The resulting plasma mixtures were analysed by the TCT test. Data represent means±sd from triplicate determinations normalized against buffer clotting times.

Based on the observation that SufA cleaves fibrinogen, we tested the effect of the proteinase on the clotting of human plasma. SufA at different concentrations was incubated with human citrate-treated plasma for 30 min, and the amount of fibrinogen available for clotting was analysed by measuring the thrombin-induced coagulation time (TCT). The enzyme was found to increase the TCT clotting time in a dose-dependent manner (Fig. 2), and at a SufA concentration above 80 nM no measurable clot was formed. To investigate if SufA influences the intrinsic or the extrinsic pathways of coagulation, clotting was measured by the activated partial thromboplastin time (aPTT) and prothrombin time (PT) respectively. SufA at concentrations causing prolongation of the TCT (0.2–40 nM) had no effect on activation of these pathways. At the highest concentration of SufA however, the aPTT and PT were substantially affected, most likely due to cleavage of fibrinogen by SufA. Thus, the data emphasize the specificity of SufA for fibrinogen resulting in inhibition of fibrin network formation.

### Generation of a *F. magna* strain lacking SufA activity

To study the role of SufA when present on the surface of *F. magna*, we constructed a *sufA* mutant by insertion duplication mutagenesis of ALB8, the strain in which SufA was originally identified. To our knowledge, there are no reports of mutants in *F. magna* or other Gram-positive anaerobic cocci, so we utilized the suicide vector pFW13 previously used for gene targeting in *Streptococcus pyogenes* (Podbielski *et al.*, 1996). pFW13 carries the *aacA*/*aphD* kanamycin-resistance gene and has a ColE1-derived origin of replication (restricted to replication in relatives of *E. coli*). For the transformation procedure, a protocol modified from a streptococcal electroporation protocol was used (Simon & Ferretti, 1991) (see Methods).

**Fig. 3. f3:**
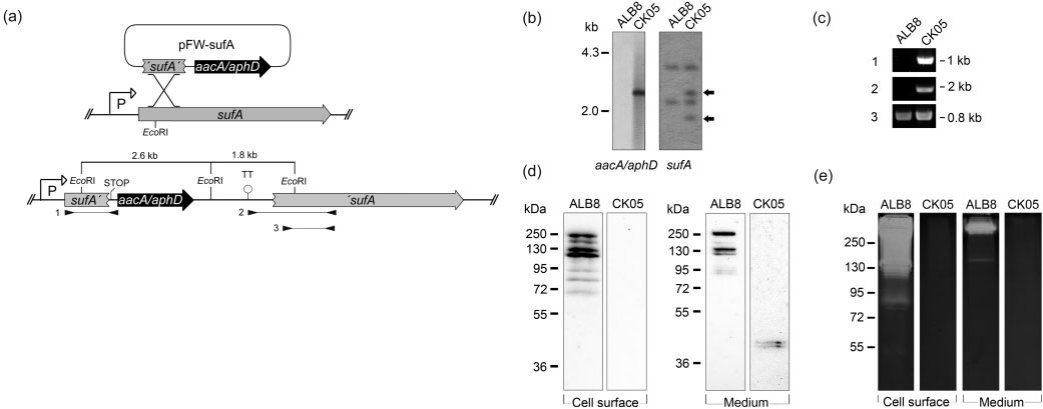
*sufA* gene disruption. (a) Schematic drawing of the strategy to disrupt *sufA* in *F. magna* strain ALB8. An internal 1010 bp fragment of the *sufA* gene (′*sufA*′) was cloned into the pFW13 vector, generating plasmid pFW-sufA. This plasmid was transformed into the ALB8 strain for homologous recombination and chromosomal integration, which resulted in the expression of a truncated SufA protein under control by the putative *sufA* promoter (P). Hairpin loop transcription terminator (TT) downstream of the *aacA*/*aphD* gene ensured silencing of ′*sufA* transcription. *Eco*RI restriction sites are indicated. The locations of the primer pairs used in PCR analysis are indicated by inverted arrowheads. (b) Southern blot analysis of *Eco*RI-digested chromosomal DNA from wild-type strain (ALB8) and the generated strain CK05 (*sufA* disrupted) separated on agarose gel. Biotin-labelled fragments of the *aacA*/*aphD* and *sufA* gene were used respectively as probes. The *sufA* probe spanned over the *Eco*RI restriction. (c) PCR analysis using genomic DNA from the ALB8 and CK05 strains as templates. The primer combinations used in the PCR are as indicated in (a). (d) Western blot analysis of crude protein preparations from strains ALB8 and CK05. Cell-surface proteins were solubilized using papain and growth medium proteins were concentrated with spin columns. The protein preparations were separated by SDS-PAGE and transferred to PVDF membranes and probed with antibodies against recombinant SufA. (e) Proteins in (d) were analysed by gelatin zymography using 8 % polyacrylamide and 0.1 % porcine gelatin. Proteolytic activities are shown as clearings in the stained gel.

An internal fragment of the *sufA* gene, covering the putative propeptide and a fragment of the putative catalytic domain, was cloned into the multiple cloning site I of vector pFW13, resulting in vector pFW-sufA (Fig. 3a[Fig f3]), which was transformed into ALB8 bacteria. The transformants were kanamycin resistant at concentrations up to 300 μg ml^−1^. One mutant clone, denoted CK05, was chosen for further analysis. To confirm vector integration and *sufA* disruption, chromosomal DNA from ALB8 and CK05 was analysed by Southern blotting, using as probes an *aacA*/*aphD* internal fragment and a *sufA* internal fragment spanning the *Eco*RI restriction site. The *aacA*/*aphD* probe only hybridized with a single band of approximately 2.6 kb of *Eco*RI CK05 chromosomal DNA digests (Fig. 3b[Fig f3], left panel). *Eco*RI digests of ALB8 chromosomal DNA exhibited two positive bands with the *sufA* probe, while *Eco*RI digests of CK05 chromosomal DNA also showed two additional bands of approximately 1.8 kb and 2.6 kb (Fig. 3b[Fig f3], right panel, indicated with arrows). For further confirmation of the plasmid integration, both end regions of the integrated plasmid and the adjacent disrupted *sufA* gene were amplified with PCR (Fig. 3a, c[Fig f3]). The PCR products 1 and 2 were DNA sequenced (data not shown); this identified two incomplete copies of the *sufA* gene. To conclude, the integration of the suicide vector yielded a targeted gene disruption of *sufA*.

When cultivated at 37 °C in an anaerobic environment, the isogenic mutant CK05 exhibited a similar growth rate to the wild-type strain ALB8 (data not shown). SufA is present at the surface of *F. magna*, but is also found in substantial amounts in the growth medium (Karlsson *et al.*, 2007). Therefore, proteins released by papain from the bacterial surface and culture medium of both wild-type bacteria (ALB8) and the mutant strain CK05 were analysed by Western blotting using SufA-specific antiserum. Several immunoreactive bands between 70 and 250 kDa were detected in papain digests of ALB8 (Fig. 3d[Fig f3]). The bands below 130 kDa, not found in the culture medium, might be SufA fragments generated by the papain digestion. No such bands were detected in the corresponding material from CK05 (Fig. 3d[Fig f3]). However, in the growth medium of this strain two protein bands of approximately 41 and 43 kDa reacted with the SufA antibodies (Fig. 3d[Fig f3]). These bands most likely correspond to the expression of a truncated SufA protein as a consequence of the plasmid integration. The theoretical molecular mass of truncated SufA containing the signal peptide is 40.3 kDa whereas the mass is 37.2 kDa without this peptide. This is in accordance with the size of the protein bands in Fig. 3(d), right panel.

SufA has been shown to efficiently degrade gelatin (Karlsson *et al.*, 2007), and the various protein preparations were also analysed by gelatin zymography. As demonstrated in Fig. 3(e), gelatinase activity was present in preparations from the wild-type ALB8 strain. No such activity could be detected in the *sufA*-disrupted strain CK05 (Fig. 3e[Fig f3]), demonstrating that disruption of the *sufA* gene results in the production of a truncated SufA protein lacking gelatinase activity.

### SufA expressing *F. magna* inhibits coagulation of human plasma

**Fig. 4. f4:**
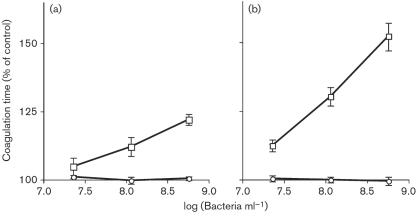
SufA-expressing *F. magna* bacteria inhibit coagulation. Human citrate-treated plasma (a) or fibrinogen solution at a concentration of 3 mg ml^−1^ (b) were preincubated with *F. magna* strains ALB8 (□), CK05 (○), or with 13 mM sodium citrate buffer for 30 min at 37 °C. Bacteria were removed by centrifugation, and the resulting plasma supernatants were analysed by the TCT test. Data represent means±sd from triplicate determinations normalized against clotting times for the buffer control.

To analyse the effect of bacteria-bound SufA on fibrin formation, the two strains ALB8 and CK05 were incubated with human plasma for 30 min at 37 °C. Following removal of bacteria, the coagulative state of the resulting supernatants was measured by TCT. The ALB8 strain prolonged the clotting times, while CK05, lacking the proteinase, had no effect (Fig. 4a[Fig f4]). The plasma supernatants were also analysed by SDS-PAGE. Cleavage products of fibrinogen, corresponding to those obtained in SufA-degraded plasma (see Fig. 1a[Fig f1]), were found in plasma samples incubated with the SufA-expressing strain ALB8. No such degradation products could be detected in plasma incubated with the mutant CK05 strain (data not shown). Fibrinogen alone was also used to monitor the thrombin-induced coagulation time. Similarly to whole plasma, SufA-expressing ALB8 prolonged the clotting time whereas CK05 bacteria had no effect (Fig. 4b[Fig f4]). At higher bacterial concentrations (≥10^9^ c.f.u. ml^−1^) no measurable clots were formed in plasma or fibrinogen solution incubated with ALB8.

### SufA produced by *F. magna* adhering to keratinocytes prevents the formation of fibrin networks

**Fig. 5. f5:**
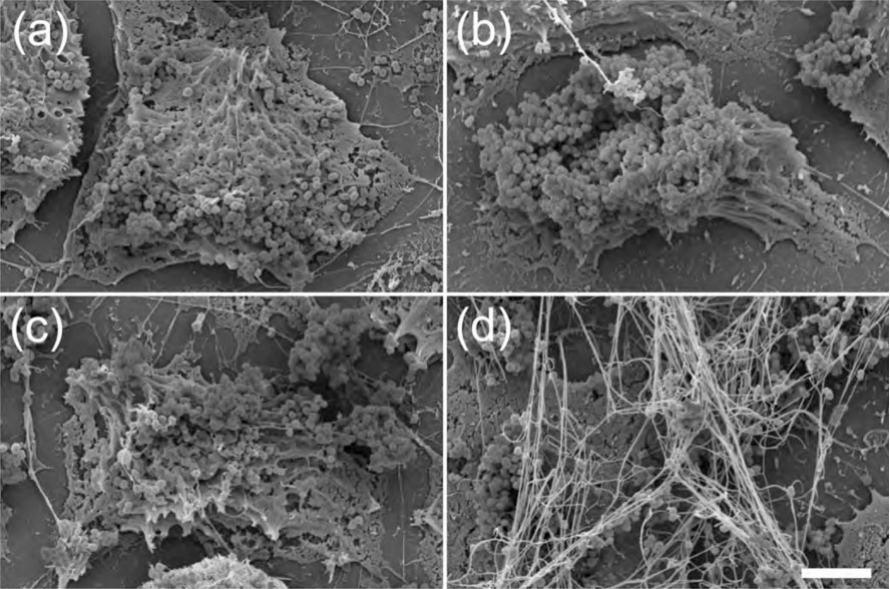
Scanning electron micographs of *F. magna* adhering to keratinocytes. Keratinocytes were incubated 2 h with ALB8 (a, b) or CK05 bacteria (c, d). The cells were then washed and incubated with buffer (a, c) or with plasma (b, d) prior to analysis by scanning electron microscopy. Scale bar: 5 μm.

**Fig. 6. f6:**
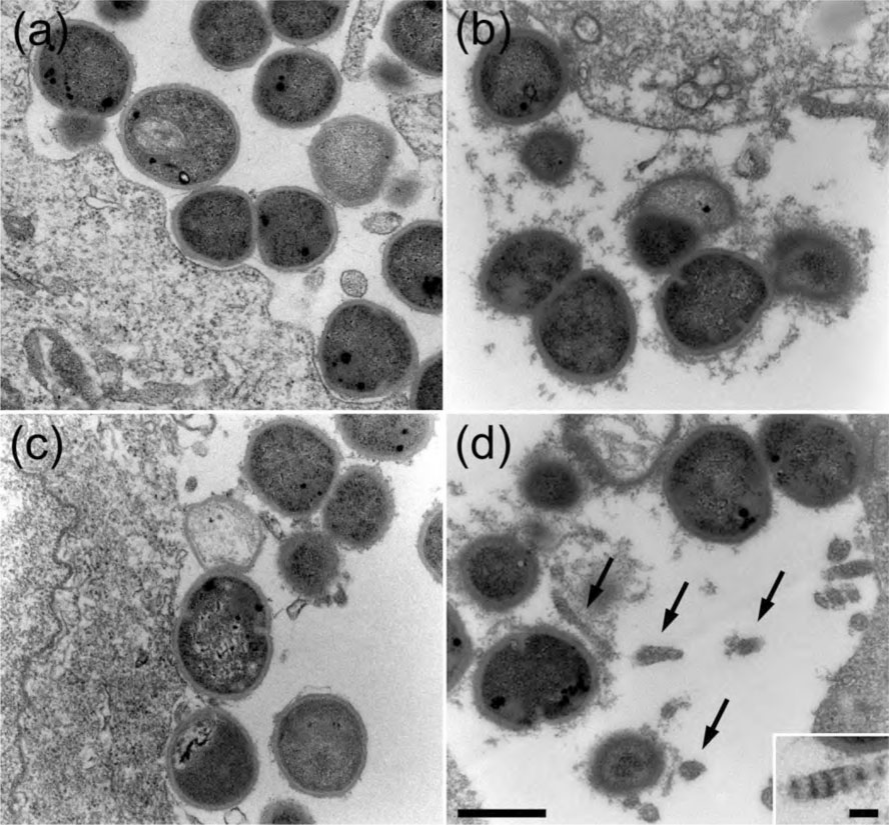
Transmission electron micrographs of *F. magna* adhering to keratinocytes. Keratinocytes were incubated 2 h with ALB8 (a, b) or CK05 bacteria (c, d). The cells were then washed and incubated with buffer (a, c) or with plasma (b, d) prior to analysis by transmission electron microscopy. Fibrin fibrils are indicated (arrows). The insert in (d) shows a higher magnification of a fibrin fibril. Scale bars: 0.5 μm (left) and 100 nm (right).

Adhesion to cells and cell matrix of the host is essential for bacterial colonization. Recent work has shown that *F. magna* in the skin adheres to BM-40 (also called osteonectin and SPARC), a protein component of basement membranes (Frick *et al.*, 2008). However, apart from basement membranes, BM-40 is widely distributed in the skin and is abundant at cell–cell contact sites of keratinocytes (Hunzelmann *et al.*, 1998), suggesting that *F. magna* may adhere to keratinocyte cell layers via BM-40. Confluent monolayers of HaCaT keratinocytes were therefore incubated for 2 h at 37 °C with bacteria of the ALB8 and CK05 strains (the expression of BM-40 by the HaCaT cells used in this study was confirmed by Western blot analysis of conditioned media and cell extracts). Following a washing step to remove non-adherent bacteria, the samples were analysed by scanning electron microscopy (Fig. 5a, c[Fig f5]) and transmission electron microscopy (Fig. 6a, c[Fig f6]). Both strains formed large aggregates and adhered equally well to the keratinocytes.

Normally, *F. magna* in the epidermis is not exposed to plasma, but in the case of wounds and inflammatory reactions (including skin infections), plasma exudate will cover *F. magna* with plasma proteins. Keratinocytes express surface-associated tissue factor (Camerer *et al.*, 1999), suggesting that fibrin networks could be formed around adhering bacteria as a result of the plasma exudation. Also, HaCaT cells express tissue factor as demonstrated by Western blot analysis of cell extracts and conditioned media. Furthermore, human citrate-treated plasma preincubated with HaCaT keratinocytes caused a prolongation of the TCT, as compared to control plasma, showing that the expressed tissue factor activates the extrinsic pathway of coagulation (data not shown). Sodium citrate-treated plasma was added to HaCaT cells with adhering *F. magna*. Following incubation for 15 min, keratinocytes and bacteria were washed and examined by electron microscopy. Bacteria of the CK05 strain, lacking SufA, were embedded by a fibrin network (Fig. 5d[Fig f5]), in contrast to the SufA-expressing ALB8 strain (Fig. 5b[Fig f5]). Transmission electron microscopy showed that bacteria of both strains are surrounded by insoluble plasma aggregates (Fig. 6b, d[Fig f6]). However, fibrin networks were only found in the vicinity of CK05 bacteria, lacking SufA (Fig. 6d[Fig f6]). The fibrils displayed periodic banding, typical for fibrin fibrils (Fig. 6d[Fig f6], inset) (Weisel & Nagaswami, 1992; for references see Wolberg, 2007). The results suggest that SufA-expressing *F. magna*, through cleavage of fibrinogen, prevents the formation of fibrin networks adjacent to bacteria adhering to keratinocytes.

## DISCUSSION

Fibrinogen is the major clotting protein of plasma, which upon cleavage by thrombin is converted into fibrin. In the present investigation, SufA, a subtilisin-like enzyme of *F. magna*, was found to modulate this target protein of the coagulation cascade and efficiently inhibit the formation of a fibrin network. In a plasma environment, soluble SufA specifically and rapidly cleaves fibrinogen, preferentially removing the C-terminal portion of the fibrinogen A*α* chains (*α*C) (see Fig. 1a and c[Fig f1]). Secondary SufA attacks the NH_2_-terminal part of the B*β* chains, and higher concentrations of the enzyme results in further processing of the A*α* chains. Fibrin assembly involves binding of thrombin to the central E domain of fibrinogen, followed by a sequential removal of the NH_2_-terminal fibrinopeptides A and B (FPA and FPB) (for references see Mosesson, 2005; Wolberg, 2007). Thrombin release of FPA is faster than the release of FPB, and delayed removal of FPA results in delayed fibrin formation. Interactions between the *α*C domains promote lateral fibril associations, clot formation and stabilization, and fibrinogen lacking these domains displays increased thrombin clotting time (Collet *et al.*, 2005; Weisel & Medved, 2001). Thus, the SufA-mediated inhibition of fibrin network formation could be explained by the removal of the *α*C domains. No clot was formed at high concentrations of SufA, most likely due to removal of central polymerization sites, where FPA and FPB are located (see Fig. 1c[Fig f1]).

Fibrinopeptides have important effects on inflammation and are, in particular FPB, potent chemotactic agents for neutrophils, macrophages and fibroblasts (Persson *et al.*, 2003; Senior *et al.*, 1986; Singh *et al.*, 1990; Skogen *et al.*, 1988). The infiltration of neutrophils will provide antibacterial peptides, such as LL-37 (Bals & Wilson, 2003), to the infectious site. It has also been demonstrated that FPA and FPB, generated upon thrombin stimulation of human platelets, exert antibacterial activity against both Gram-negative and Gram-positive bacteria (Tang *et al.*, 2002). The level of FPA in plasma treated with SufA was, however, similar to the control (data not shown), suggesting that released fibrinopeptides are further degraded and inactivated by the enzyme. Thus, SufA cleavage and inactivation have been shown for other antibacterial peptides, such as LL-37 and the chemokine MIG/CXCL9 (Karlsson *et al.*, 2007). It is also conceivable that the fibrinogen fragments generated by SufA cleavage are antibacterial. In this context, it could be speculated that such fragments preferentially are directed against pathogenic organisms, favouring growth of commensal bacteria such as *F. magna*. Alternatively, SufA-released fibrinogen fragments could block the activity of antibacterial peptides, as was demonstrated for glycosaminoglycans released from proteoglycans degraded by bacterial proteinases (Schmidtchen *et al.*, 2001).

In addition to their haemostatic function, fibrinogen and fibrin play important roles in wound healing, where the fibrin network provides a temporary matrix in which cells can proliferate during wound repair (Drew *et al.*, 2001; Laurens *et al.*, 2006). Several cellular interactions with fibrin(ogen) occur through binding to integrins via RGD sequences located in the *α*C domains. Apart from its role in fibrin assembly, the fibrin B*β*15−42 sequence promotes fibroblast proliferation, platelet spreading and endothelial proliferation (Laurens *et al.*, 2006). In addition, fibrinogen interacts with the inflammatory cytokine interleukin-1*β*, enhancing its activity (Sahni *et al.*, 2004) and binds to fibroblast growth factor-2, platelet-derived growth factor, and vascular endothelial growth factor. Fibrinogen in these complexes thereby also potentiates cell proliferation and migration (Rybarczyk *et al.*, 2003; Sahni & Francis, 2000). Presumably, the cleavage of fibrinogen by SufA will modulate not only fibrin polymerization, but also other fibrinogen-mediated functions of importance during inflammation and wound healing.

SufA is both secreted and associated with the surface of *F. magna* (Karlsson *et al.*, 2007). The soluble SufA used in this study was purified from the surface of ALB8 bacteria and despite several purification steps, including ion-exchange chromatography and gel filtration, the preparation was heterogeneous. SufA forms dimers/multimers, which are more proteolytically active than the monomeric form, and preparations of the enzyme are in general heterogeneous (Karlsson *et al.*, 2007). In Western blot analyses of the protein preparation, bands compatible with the size of monomers and dimers of both the mature SufA and the pro-enzyme react with antibodies raised against recombinant SufA (see Fig. 1e[Fig f1]). Like soluble SufA, whole bacteria were found to cleave fibrinogen and delay clot formation in human plasma. Also, on the surface of human keratinocytes adherent SufA-expressing *F. magna* displayed anticoagulative properties. In contrast, fibrin networks were formed around mutant bacteria expressing a truncated and inactivated form of SufA. The possibility of polar effects on transcription of downstream genes in the CK05 mutant strain cannot be completely excluded. To confirm the entire role of SufA would require complementation of the gene. However, the cleavage of fibrinogen and delay of clot formation obtained with soluble SufA demonstrate that the enzyme exerts potent effects on the formation of fibrin networks. The host response to a bacterial infection or tissue damage often results in local activation of coagulation and thrombus formation, providing a barrier against bacterial spread. However, several bacterial species have evolved mechanisms to circumvent this host defence by the expression of proteins activating plasminogen into plasmin, the host's clot-dissolving proteinase (for references see Sun, 2006). For instance, the human pathogen *Streptococcus pyogenes* secretes streptokinase, which was found to be a key virulence factor of this Gram-positive bacterium, enhancing invasive infection in transgenic mice expressing human plasminogen (Sun *et al.*, 2004). Thus, to dissolve fibrin clots facilitates establishment of infection, and this mechanism plays an important role for many invasive pathogens (Sun, 2006).

*F. magna* is a commensal bacterium that inhabits the skin and other non-sterile body surfaces of the human host, but it is also an important opportunistic pathogen (Murdoch, 1998). *In vitro* SufA is already expressed in the early exponential growth phase (Karlsson *et al.*, 2007). However, *in vivo* the expression of SufA might be regulated to meet the demand for bacterial survival and proliferation. During inflammatory conditions upregulation of SufA and inhibition of fibrin polymerization would favour bacterial spread to deeper tissue sites, whereas bacteria within a fibrin network could be protected from innate immune mechanisms such as phagocytosis and attack by antibacterial peptides. To be able to manipulate and modify fibrin clotting will presumably add selective advantages to *F. magna* in the normal flora, but may also promote virulence in cases of clinical infections with this opportunistic pathogen.

## Figures and Tables

**Table 1. t1:** SufA-cleaved fragments of plasma proteins and fibrinogen identified by MALDI-TOF

Sample*	**Protein**	**Accession no.†**	**Peptide match‡**	**Coverage (%)§**	**Peptide residue match||**		**Expected value**
					**Start**	**End**	
1	FIBA_HUMAN	P02671	14	10	72	271	0.00066
2	FIBA_HUMAN	P02671	10	9	39	271	0.045
3	FIBA_HUMAN	P02671	19	17	49	287	8.3×10^−5^
4	FIBA_HUMAN	P02671	14	13	49	287	2×10^−8^
5	FIBB_HUMAN	P02675	26	47	164	491	1.6×10^−12^

*Protein bands: 1, plasma 35 kDa; 2, plasma 32 kDa; 3, fibrinogen 35 kDa; 4, fibrinogen 32 kDa; 5, fibrinogen 58 kDa. †Swiss-Prot accession number. ‡Number of tryptic peptides matched to the protein. §Percentage of protein sequence covered by the matched peptides. ||Location of tryptic peptides in protein sequence.

## Abbreviations

- *α*C, C-terminal domain of fibrinogen A*α* chain
- CDS, coding sequence
- FPA, fibrinopeptide A
- FPB, fibrinopeptide B
- SufA, subtilase of *Finegoldia magna*
- TCT, thrombin-induced coagulation time
